# Sweating the Details: An Interview with Jamie Thomson

**DOI:** 10.1371/journal.pgen.1000182

**Published:** 2008-08-29

**Authors:** Jane Gitschier

**Affiliations:** Departments of Medicine and Pediatrics, Institute for Human Genetics, University of California San Francisco, San Francisco, California, United States of America

**Image 1:**
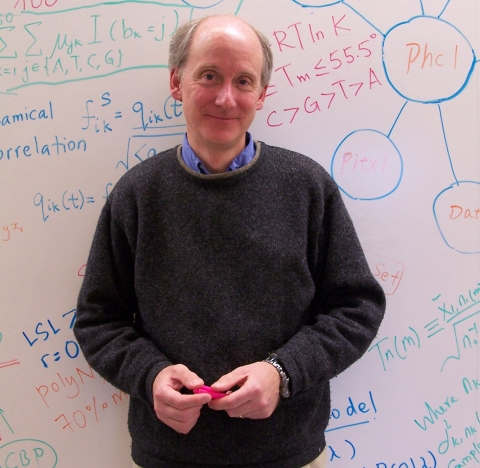
Jamie Thomson

If you had to name the most controversial scientific achievement of the past decade, you'd be hard pressed to top the development of human embryonic stem [ES] cells. Human ES cells followed on the heels of another major technological advance—Dolly, the cloned ewe. Together, these remarkable breakthroughs have stimulated great public interest and have ushered in a new era in the exploration of human biology. At the center of the ES maelstrom is a soft-spoken and intensely private scientist from the Genome Center at the University of Wisconsin. Jamie Thomson (Image 1), who is also Director of Regenerative Biology at the new Morgridge Institute for Research and the founder of two companies, is purposeful, with an obvious knack for a difficult experiment, yet seems a bit uncomfortable in the limelight his work has generated.

Trained as a veterinarian and research scientist, Thomson had a dual passion for experimental embryology and species preservation. He was just emerging from his post-doctoral fellowship at the Primate Center in Oregon when he moved to Wisconsin. He was hired there with the specific goal of deriving ES cells from primates, a feat he accomplished in short order. Within a few years, working closely with the ethics and in vitro fertilization (IVF) communities, he succeeded in deriving human ES cells. More recently, pushing the boundaries of human developmental biology even further, his laboratory reported the creation of induced pluripotent stem (IPS) cells, a way for turning human differentiated cells back into ES cells.

I sought out Jamie in mid-April during a cold, umbrella-inverting rainstorm. With daughter in tow, I borrowed my sister's red MINI Cooper and headed off from her home in Milwaukee to Madison. After struggling with the concept of paying for parking by phone, we puddle-jumped our way to Thomson's building and literally stumbled into a sign celebrating the first synthesis of DNA, one of a series of placards that extol the long line of important discoveries made at this University. I located Thomson in his office down a quiet hallway, abandoned my daughter to the tea room and her assigned reading in *To Kill a Mockingbird*, and to the soothing sound of the University of Wisconsin coal train outside his window, we began the interview.

**Gitschier**: The first thing I want to talk to you about is how you got into this business.

**Thomson**: In college, I liked biology, and I wasn't quite sure what I wanted to do with it. I was good at mathematics. I was trying to find a way to put the two of them together in a biophysics major. But I was spending a summer at Woods Hole, doing population biology stuff, because I thought that was a good overlap with understanding mathematics.

**Gitschier**: What kind of organism were you working on?

**Thomson**: I was working on the salt marshes with *Melampus bidentatus*, which is a little snail. And I was up to my knees in mud the whole summer in the Great Sippewissett Marsh, and I decided that I liked mathematics, but I wasn't really into counting things that large. So I was looking around for a way to put these things together, and I liked developmental biology still.

One night, I went to a lecture by a guy from Stanford at the time. His name was Paul Ehrlich, an ecologist type, and he gave a talk on endangered species. He quickly went through this argument that zoos would have no positive impact [on preventing extinction] because of the size and the numbers [of endangered species] involved. If you filled up the zoos with that number, you'd hit only a very small number of species, and you're not going to do anything significant.

I was sitting in the audience going “Hmm, I thought you could freeze things like sperm and eggs.” I was a biophysics major, what did I know? But I thought that if you went out and collected enough sperm and enough eggs, you could keep fairly large repositories and the breeding problem could be managed artificially through this stock of frozen stuff.

That same evening on the news there was a woman named Barbara Durant with Walter Cronkite at the San Diego Zoo, and she had this rat that had come from a frozen embryo.

So all of this happened in one day! And I looked at that and said, “I'd like to do that! I wonder what kind of degree would be useful for that?”

**Gitschier**: What year was this?

**Thomson**: This would have been 1980, between my junior and senior year.

I had an undergraduate advisor named Frederick Meins who, even though I had a very good grad school advisor, has been my mentor in life. When I was 18, he told me all these wonderful stories about these biologists, one of whom was Jared Diamond, who would be on the cover of the *New York Times* for discovering some new species in New Guinea. But he was also a biophysics professor! And I thought, “Hey, I could do something like that!”

So the plan was, in my naive undergraduate ambition, to do practical veterinary things that would allow me to manipulate embryos of endangered species, and at the same time, do basic developmental biology. It turned out that Penn had a combined VMD/PhD program. So, I went to graduate school there in a basic mouse developmental biology lab [with Davor Solter] at the Wistar Institute.

When I went to be a post-doc, I decided to switch to primates, to go back to this original idea. There are about 200 species of primates, depending on whether you are a lumper or a splitter, and at that time, about half were endangered or threatened. And it seemed like a focused population with biomedical relevance. And if I did basic developmental biology in primates, it would be directly applicable to humans in a way that mouse is not.

So I went off to the Oregon Primate Center, which at that time had the best embryo recovery and in vitro fertilization [IVF] program for nonhuman primates. I learned a lot about doing IVF and experimenting with primate embryos and, at the same time, I initiated some fieldwork in Sulawesi [in central Indonesia] with an endangered primate called *Macaca nigra*. They had gone from 300,000 in the '70s down to about 3,000 by the time I got there. I was going to attempt to dart the remaining male population, collect semen, and bring it back home—some preliminary stuff to get a grant. I think the grant came in second and they [the NIH] decided to fund only one. So I started looking around for a job, because things were ending in Oregon.

I moved here [Wisconsin] because John Hearn was the director of the Primate Center here, and he had worked out methods to have in vivo–recovered embryos. He hired me specifically to derive primate embryonic stem cells.

**Gitschier**: Were these embryos the product of in vitro fertilization?

**Thomson**: No, these were natural ones, flushed out in a nonsurgical procedure. We got very high-quality embryos that don't require culture. The advantage [of using natural embryos] was that back then, the culture medium [for IVF-generated embryos] was pretty bad, and you couldn't go from one cell to blastocyst and have a real healthy product.

So I came here specifically to derive primary embryonic stem cells.

**Gitschier**: But wait, is this a leap? You were interested in saving embryos that could then be used to regenerate lost species, potentially.

**Thomson**: No, the intent was to establish a robust experimental embryology in primates that could make up for some of the species-specific differences in mouse. Mouse is simply a better model, there is no way around that, but in some ways it does not reflect human development very well. And in Oregon, despite the fact that they had the best embryo recovery program from IVF in the world, they were completely starved for embryonic material.

In mouse, you can sit down and do 200 embryos in a day—it's not a big deal. The cost here for the natural flushed [primate] ones was $2,000 per embryo! So you couldn't do a thousand of them, you couldn't do two of them! It was almost impossible.

So, the rationale for me to derive the primary embryonic stem cells was to get a sustainable primate material that would recapitulate normal human events better than mouse ES cells in a way that the material is not limiting.

**Gitschier**: OK!

**Thomson**: It's all about experimental embryology. And Hearn had been Director of the research branch of London Zoo. He had a strong interest in promoting conservation of primates when he came here. And I still had this idea that I could do both [experimental embryology and conservation], until I derived embryonic stem cells, and then it kind of took over my life.

Since 1995, when we published the first rhesus embryonic stem cells, those other interests in endangered species have been pushed aside.

**Gitschier**: Would you like to get back to that sometime?

**Thomson**: Part of me wants to. I have a young family now. Mucking through the jungle is not going to happen any time soon. Although it is attractive and I would probably enjoy doing that more than what I'm doing now.

**Gitschier**: Let's talk about 1995 and your work on primate embryonic stem cells. It sounds as though it worked fairly quickly. What did you do that was different from the mouse?

**Thomson**: It was sweating the details. Superficially it was the same. The primate cells are not dependent on LIF [leukemia inhibitory factor], but feeder layers worked also for these cell lines. We were very careful with the culture conditions and got it to work. Later on, we and others discovered that cytokines that mediate self-renewal are distinct between the two cell types, but we didn't know it at the time.

**Gitschier**: Why had no one done it before? Mouse ES cells were derived in 1981. Fourteen years later…

**Thomson**: In the middle 1980s, people in Britain had already tried to do human ES cells and failed. And failed probably because the embryos weren't very good, because the culture medium was bad, and they were still thinking they were like mouse ES cells, which they are not. Even though the conditions we use involve fibroblasts, as in mouse ES cells, the finesse is different, the timing is different, the splitting is different. Some very good mouse ES cell people attempted to do this, and I don't know why they failed, but they did fail.

We had good access to very, very high-quality primate material. We were very quickly successful with that, and because we had that experience, as soon as we had high-quality human embryos, we got it. If you look at the numbering of our cells lines, it goes from H1 to H14. H1 is the very first human embryo we tried, because of our experience with primate. And the culture medium was just getting better at the time we were developing our cells.

**Gitschier**: What was different about the medium?

**Thomson**: David Gardner, working in Australia and now in Colorado, developed a new generation of media; it was optimized for human material. If you put mouse media on human embryos, it doesn't work very well. It's nothing to do with a special growth factor, it's just optimizing salts and glucose, stuff like that.

**Gitschier**: So it's a medium for the embryos themselves, not the stem cells.

**Thomson**: Right. Prior to that we couldn't get a quality blastocyst to try it [stem cell derivation] on. With the primate work, we didn't need the medium because we went straight to the derivation process, because the [in vivo–recovered] embryos were high quality.

**Gitschier**: So were you communicating with the people who do IVF and who actually culture the embryos?

**Thomson**: Oh yeah. Jeff Jones was the person who actually did that and Gardner had a graduate student who became a post-doc at UW, and that post-doc helped Jeff introduce that medium into the IVF clinic. So this clinic was among the first to use that new generation of media. Part of the reason we were successful is that Jeff is very good at his job.

**Gitschier**: So, 1995, you were still interested in primates for primates' sake, really, looking at early embryonic development. Why did you then make the leap to humans?

**Thomson**: At the time we derived the primate stem cells, I really wasn't planning to do it. I assumed that once we published the primate work, someone would do the human work very quickly. We have a very small IVF unit here, with very limited access to embryos, and I thought that every IVF lab in the world would be doing this quickly.

**Gitschier**: And presumably, it wasn't even a primary interest of yours.

**Thomson**: No, though it was obvious that it was really important. I just thought someone else would do it. But as the months wore on and nobody did it, I decided to try it here.

**Gitschier**: And did you do this with your own hands?

**Thomson**: Yeah, I did all those experiments.

At the end of 1994/95, I spoke to our ethics people here. In 1994, Clinton asked [Harold] Varmus [then director of the NIH] to start a commission to look at embryo experimentation and Alto Charo, who is a lawyer here, sat on that panel. So I asked her, “Hey, if I were to do this, what should I do?” I was really lucky that she was here. Also, the head of our IRB, Norm Fost, was very supportive.

Things have basically been “won” now, from a public perception. But back then, everybody was really scared. They were scared about public funding. They were scared about personal safety. While the University wasn't ecstatic about having me around, because it made everybody nervous, they were all really supportive, and in particular those two people helped me put the consent process through in a very reasonable way.

**Gitschier**: Did you personally have any ethical issues with the human embryonic stem cells?

**Thomson**: I thought about it a lot prior to doing it, and I decided that if these are embryos that the patients have already decided to throw out, that it is a better ethical choice to use them for something useful. And it's actually a fairly complex problem about how you think about that.

**Gitschier**: At the time was there already some kind of prohibition to funding human embryonic research?

**Thomson**: Yeah, it was actually worse then. The Dicky amendment [1996] said something to the effect that no federal money can be used to damage or jeopardize a human embryo.

**Gitschier**: So in addition to getting the IRB approval, you also had to get money.

**Thomson**: Right, and that's what made the University so nervous, because they would lose all the federal funding if I screwed up somehow.

**Gitschier**: No pressure! Is that when you started to be supported by Geron [Corporation]?

**Thomson**: I asked the University for about $20,000, and they said “No.” And the next week Geron walked into my office and I said, “Well if the Federal government won't fund me and the University won't fund me, great!”

Mike West was the fellow at Geron at the time. He's got the vision thing down. He understood that this was important before other people did. I accepted funds from Geron until President Bush made it legal to use federal funds, and I have declined them ever since.

**Gitschier**: But you need to use existing cell lines.

**Thomson**: I've used the same five cell lines for 10 years now.

**Gitschier**: You made a comment in another interview: “I didn't know we'd be stuck with these cells.”

**Thomson**: Yeah, I assumed people would derive new ones right away.

**Gitschier**: But the implication is that these early embryonic stem cells might not be as good as ones that might be derived later.

**Thomson**: Actually, to be stronger than that, I very specifically did not keep track of stuff, like lot numbers, because I didn't want to have to do the paperwork for FDA-like stuff. I figured, this is nice proof of principle. We'll just derive MORE! If people want to do a GMP [good manufacturing process], let them. The FDA actually wants tracking now.

**Gitschier**: Let's talk about how your life changed then in 1998 with the publication of human stem cells.

**Thomson**: Well, Dolly had been cloned the year before, so I was kind of prepared, because I could see what Ian Wilmut had to go through.

**Gitschier**: And what did he have to go through?

**Thomson**: Hell. It doesn't allow you much time for doing your work anymore.

So I was wrong about some things. For one thing, the media were pretty positive, but it wasn't clear prior to publication how it would go. On the whole, the initial science reporting especially was superb and the story was accurate.

The other thing I miscalculated was that I figured people would have a pretty short attention span. But we're 10 years in, and it is still a big story. I think it has to do with politics and who got elected to the White House more than anything, because I think had somebody else been elected, it [stem cell research] would have been “normal” science a long time ago.

**Gitschier**: And now, you've developed a new technology that may obviate some of these political and funding issues: Induced pluripotent stem [IPS] cells. Set the stage for that for me.

**Thomson**: The stage was Dolly really—that changed the mindset of developmental biologists in a big way, including mine. About 5 years ago, I hired the post-doc [Junying Yu] who was the first author on our paper [published in 2007]. My conversation with her at the time was that we have to try this, even though it probably isn't going to work. And it's probably like a 20-year problem, because the thought back then was that it has just got to be really complicated. All those little factors, and how can you manipulate all of those? It didn't really seem sensible.

I thought by doing such a combinatorial screen, we might get PARTIAL reprogramming in some way.

**Gitschier**: Describe what you mean by combinatorial screen.

**Thomson**: I'll tell you what we did, and it was very similar to what Yamanaka did in the mouse. We were doing it at the same time, but he got ahead of us because mouse work is actually much faster than human work, although we actually had a partially defined system with a more complicated set of factors prior to publication of his mouse work.

Back in the '70s, it was found that if you fuse blood cells with embryonic carcinoma [EC] cells—ES cells hadn't been derived yet—that within that heterokaryon, the dominant phenotype could either be the blood cell or the EC cell, but it was often the EC cell. So that was early evidence for reprogramming.

We started to do similar experiments several years ago, in which we took ES cell–derived blood cells. We had a well-defined, cloned, expandable hematopoietic cell type that we used in cell fusions for a model for reprogramming, and we showed that the dominant phenotype was the ES cell.

We did gene expression analysis of both those cell types and started to clone genes that were specifically enriched in ES cells. So Junying cloned between 100 and 200 genes, and she started taking pools of them to test for reprogramming ability and we used a knock-in human ES cell line that turns green and gets drug resistant when it reprograms to an ES cell state. Last summer, Junying kept paring it down until there were four factors, and we repeated it in different cell types.

It was kind of a dumb thing to do—it worked and that is nice. If you look at the factors we found, *OCT4*, *SOX2*, and *NANOG*—they're everybody's favorite genes already—these are key pluripotency genes. But we had this mindset, which was so strong, that it HAD to be complicated, we just never tested them! It would have been a lot easier to just test them 5 years ago and gotten it done in a month or two!

**Gitschier**: These IPS cells won't be restricted in terms of federal funding?

**Thomson**: No. That changes everything.

**Gitschier**: Do you think people will work both on IPS and ES cells?

**Thomson**: For our lab, we've been growing the same ES cells for 10 years—we're not going to stop that anytime soon. On the other hand, as new people enter the field, I would guess that most of them will be deriving IPS cell lines and not ES cell lines, and that over time, ES cells will take a smaller percentage of people's attention. It comes down to whether they are equivalent or not, which we don't know for sure yet. But my sense is that they will be. If you can't tell them apart, why inherit all the baggage of the embryonic stem cells when you don't have to?

**Gitschier**: So, what are you going to work on now?

**Thomson**: I'm interested in whether this is the first example of many. There are other cell fusions between other cell types, and it suggests that other similar lateral transitions could be artificially induced. Nobody has done systematic screens for those lateral transitions in a similar way, probably because everybody thought that was too complicated. There is probably no clinical utility for making a heart cell out of nerve cell, but that is the kind of thing we're going to see. Can we understand enough about the biology that we can predict which factors are needed without doing these combinatorial screens? I don't know precisely how you get this into regenerative medicine, but when you actually think about the nuts and bolts of how you'd introduce something into a patient, that is challenging.

The long-term goal of regenerative medicine is to cause tissue to regenerate, not to do cell transplants. And for tissues to regenerate that don't normally do so, you're talking about changes in cellular states that are physiologically disallowed. So if you understand what creates those barriers and how you can overcome them, it could well lead to real robust regenerative medicine. And that one is DEFINITELY a 21st century problem that won't go away as fast as the last ones.

**Gitschier**: Are you having fun?

**Thomson**: Yes and no. It's very satisfying—that what I do I think is important. But day to day, most jobs are just stressful, you know? Even if you are good at it, and things are going well.

**Gitschier**: Do you think that part of the stress, though, is the nature of the problem itself and the competition?

**Thomson**: Yeah, the competition has gotten—I won't say out of hand—that is the nature of the business—if something is perceived as important, there will be competition, but it clearly makes it a lot less fun, and I don't see a way around that. Especially the reprogramming stuff over the last year or so—everybody is doing that now! That was one of nice things about doing the primate ES cells—nobody cared!

By nature, I am a loner. At the last ISSCR [International Society for Stem Cell Research] meeting, I don't know how many people show up now, but based on my personality, too many! I do better one on one or in small groups.

**Gitschier**: But this is a path you're going to be on for a while.

**Thomson**: Yes, and I'm looking for little niches that I can find fun again for which there is not a head-to-head competition, ‘cause at the end of the day, if you publish a week or two before somebody else, it's kind of futile, isn't it?

**Gitschier**: Final thoughts?

**Thomson**: The point that I want to make is that a lot of the enthusiasm and emotion that has driven human embryonic stem cells is the idea that we'll use this for transplantation and cure diseases like Parkinson, and while I think in limited cases that might be true, I think broadly it will prove extraordinarily challenging. And if you look at the field say 10 to 20 years from now, there'll be some stellar successes in the transplantation realm, but there'll be a lot of failures, and I think people are ill-prepared for the risks of a new technology like this.

If you look at the early days of bone marrow transplants, most people died! I think a single death in this area is going to create an uproar, given the politics involved, and there will be such deaths, because the diseases people are contemplating [treating] are very serious diseases.

So if you look at the pie of things that will be important 20 years from now, my belief is that the broader applications are in the understanding of the human body, and that, similar to recombinant DNA, it will be pervasive and everybody is going to use it and they won't call themselves “stem cell biologists” anymore. It'll just be something to get access to the human body. And I think that WILL profoundly change human medicine in ways that I can't even predict.

